# Cross-platform mobile app development for disseminating public health information to travelers in Thailand: development and usability

**DOI:** 10.1186/s40794-022-00174-6

**Published:** 2022-07-15

**Authors:** Pongthep Meankaew, Saranath Lawpoolsri, Watcharapong Piyaphanee, Peerawat Wansatid, Pimwadee Chaovalit, Siam Lawawirojwong, Jaranit Kaewkungwal

**Affiliations:** 1 Department of Tropical Hygiene, Faculty of Tropical Medicine, Mahidol University, 8th Floor and 9th Floor Tranakchit Harinasuta Building, 420/6 Ratchawithi Road, Ratchathewi, Bangkok, 10400 Thailand; 2 Center of Excellence for Biomedical and Public Health Informatics (BIOPHICS), Faculty of Tropical Medicine, Mahidol University, Bangkok, Thailand; 3 Thai Travel Clinic, Hospital for Tropical Diseases, Faculty of Tropical Medicine, Mahidol University, Bangkok, Thailand; 4 The National Electronics and Computer Technology Center (NECTEC), Pathum Thani, Thailand; 5 Geo-Informatics & Space Technology Development Agency (public organization), Bangkok, Thailand

**Keywords:** Cross-platform, Mobile app, Travelers, Disease surveillance

## Abstract

**Background:**

The risk of disease is a key factor that travelers have identified when planning to travel abroad, as many people are concerned about getting sick. Mobile devices can be an effective means for travelers to access information regarding disease prevalence in their planned destinations, potentially reducing the risk of exposure.

**Methods:**

We developed a mobile app, ThaiEpidemics, using cross-platform technology to provide information about disease prevalence and status for travelers to Thailand. We aimed to assess the app’s usability in terms of engagement, search logs, and effectiveness among target users. The app was developed using the principle of mobile application development life cycle, for both iOS and Android. As its data source, the app used weekly data from national disease-surveillance reports. We conduced our study among visitors to the Travel Clinic in the Hospital for Tropical Diseases, Faculty of Tropical Medicine, Mahidol University, Bangkok, Thailand. The participants were informed that the app would collect usage and search logs related to their queries. After the second log-in, the app prompted participants to complete an e-survey regarding their opinions and preferences related to their awareness of disease prevalence and status.

**Results:**

We based our prototype of ThaiEpidemics on a conceptualized framework for visualizing the distribution of 14 major diseases of concern to tourists in Southeast Asia. The app provided users with functions and features to search for and visualize disease prevalence and status in Thailand. The participants could access information for their current location and elsewhere in the country. In all, 83 people installed the app, and 52 responded to the e-survey. Regardless of age, education, and continent of origin, almost all e-survey respondents believed the app had raised their awareness of disease prevalence and status when travelling. Most participants searched for information for all 14 diseases; some searched for information specifically about dengue and malaria.

**Conclusions:**

ThaiEpidemics is evidently potentially useful for travelers. Should the app be adopted for use by travelers to Thailand, it could have an impact on wider knowledge distribution, which might result in decreased exposure, increased prophylaxis, and therefore a potential decreased burden on the healthcare system. For app developers who are developing/implementing this kind of app, it is important to address standardization of the data source and users’ concerns about the confidentiality and safety of their mobile devices.

## Background

Several studies have reported that mobile technology use in everyday life has an impact on lifestyle, and business including education, communication, social networking relationships, health care, data transmission, disease surveillance, medical practice, and chronic disease management [1–5]. Information from disease surveillance systems is useful for reporting and monitoring public health. A main goal of disease surveillance reporting systems is disseminating, communicating, and providing access to such information regarding a population’s state of health.

Surveillance has been defined as the “ongoing systematic collection, analysis, and interpretation of outcome-specific data for use in the planning, implementation, and evaluation of public health practice” [6]. There are many different channels for presenting disease surveillance information to the public. Disseminating through mobile channels is one of the most interesting trends for information reporting, as individuals expect to be able to access information and services on their mobile devices at any moment [7]. Thailand has been operating a disease surveillance system using the report card R506 for many decades. R506 is a national disease surveillance tool to collect, manage, and analyze health data; it uses processed information to monitor the health situation and status of the national population and is managed by the Bureau of Epidemiology (BoE) of the Ministry of Public Health The BoE is the center for data warehousing, and it receives data from hospitals and health centers across the country. On a weekly basis, government hospitals and health centers in Thailand submit data to the R506 system. The reported data are processed, analyzed, and published on the BoE website every week. However, the BoE’s R506 reports derive from the website, which fits the large screens of laptop and desktop computers. The reports cannot be properly displayed on mobile devices, though several surveys have shown that mobile apps are more popular than Web-based ones owing to greater usability, convenience, and speed [8, 9].

As noted above, one of the most important aspects with a disease surveillance system is dissemination: the system should appropriately display information, deliver it to the target audience, and facilitate information finding [10, 11]. As in many countries, the Thai economy relies on tourism. Prior to the COVID 19 pandemic in late 2019, there were 35.6 million and 37.2 million tourists to Thailand in 2017 and 2018 respectively [12]. In 2018, Thailand was one of the top 10 countries for receiving the highest number of tourists [13]. Since the COVID-19 outbreak, tourism in Thailand during 2018-2019 has drastically declined, and halted during lockdown periods from 2020 to present. Several apps created by Thai government agencies help travelers for specific purposes; examples include informing travelers about exchange rates, weather, and tourist police details. However, there has been a lack of apps providing tourists with information regarding disease related details specific to areas of travel. With the reopening of the country in mid 2022, the dissemination of such information would become even more valuable for tourists who plan to visit Thailand.

Global use of mobile devices has grown rapidly since 2014, covering multiple platforms, such as Android, iOS, and Windows [14–18]. Over 100,000 health-related apps have been developed for the iOS and Android platforms [19]. Cross-platform development remains the greatest challenge for mobile app developers as they have to provide the same app with a single coding set that can run on multiple operating systems. The app should also be compatible with native mobile apps, which are created and run only on the platform for which they were designed.

The main purpose of the present study was to develop a cross-platform mobile app, called ThaiEpidemics. The app was based on the conceptualized framework for visualizing R506 data for tourists who visited Thailand. We developed a prototype of the app, which we tested and evaluated in terms of its usability among target users.

## Methods

### App development process

We developed the ThaiEpidemics app based on the mobile application development life cycle (MADLC) model [20, 21]. In creating the app, we obtained information about user requirements through in-depth interviews with three experts working at the Travel Clinic at the Hospital for Tropical Diseases, Faculty of Tropical Medicine, Mahidol University, Bangkok, Thailand. Since opening in 2004, the clinic has serviced thousands of foreign travelers annually, providing healthcare services, vaccination, and consultation. We collected details concerning user requirements for the app prototype from five travelers who visited the clinic. We also collaborated with the director and individuals responsible for the surveillance databases of the Department of Disease Control, BoE. We developed ThaiEpidemics using React Native (a framework for JavaScript, and cross-platform technology). We selected React Native because it is an open-source software for building cross-platform apps that utilizes a single industry standard language.

### App design and development

**Fig. 1:**
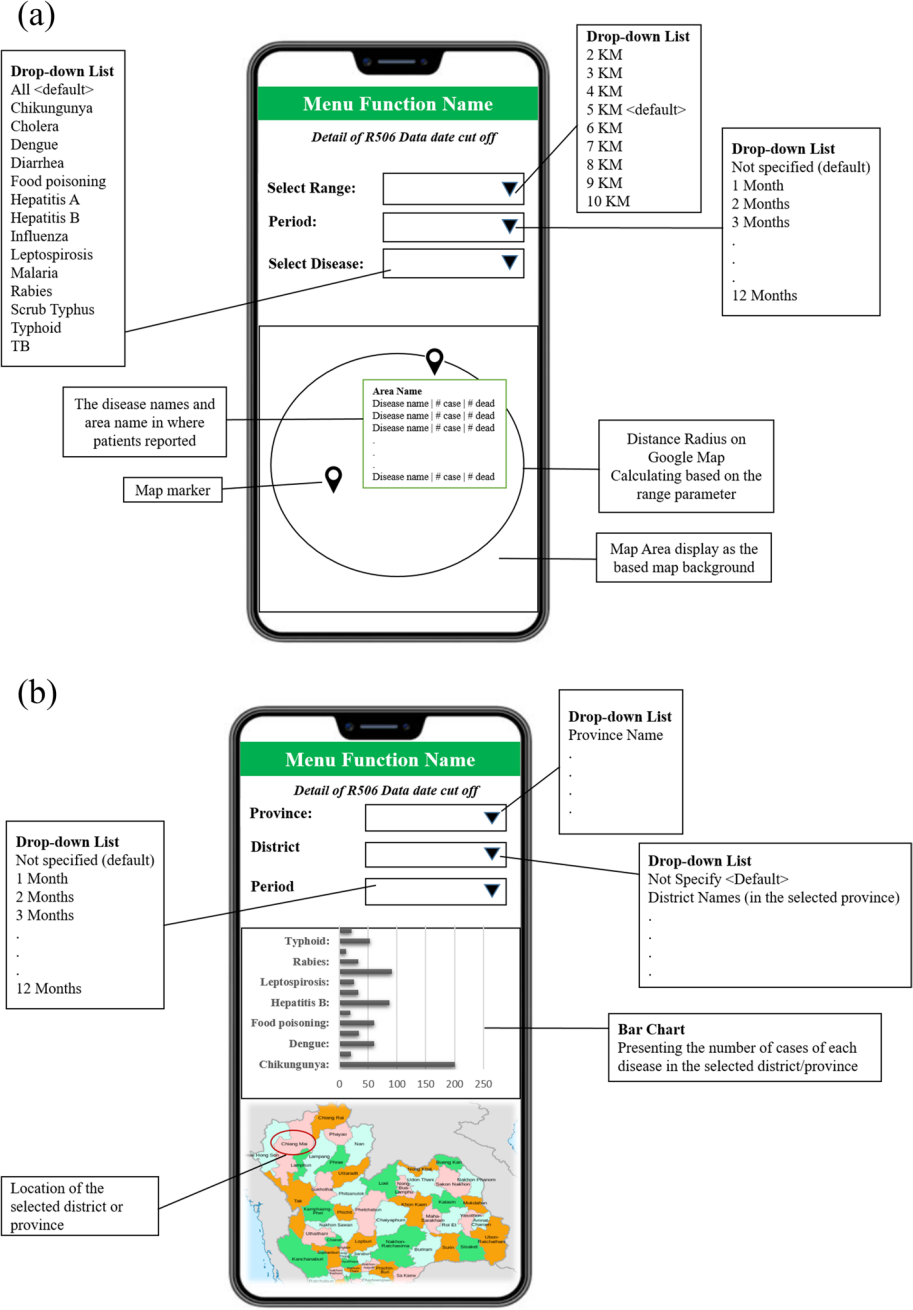
Design of the user interface in ThaiEpidemics based on main user requirements: (**a**) visualizing the disease prevalence and status for the current location; (**b**) visualizing the disease prevalence and status for a specified area

**Fig. 2:**
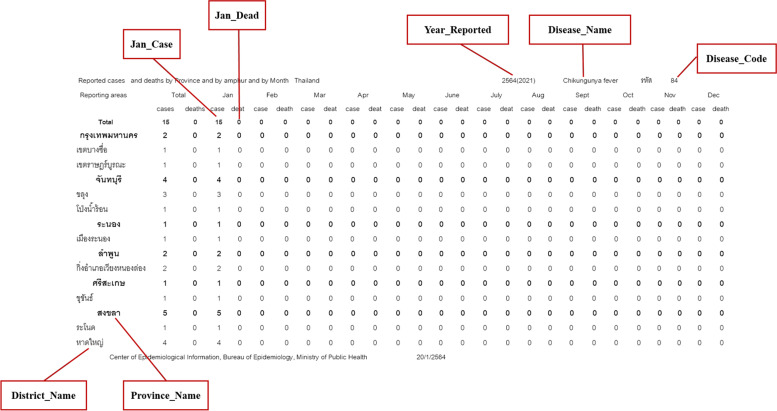
Screenshot of an R506 report and field mapping data for the database

**Fig. 3:**
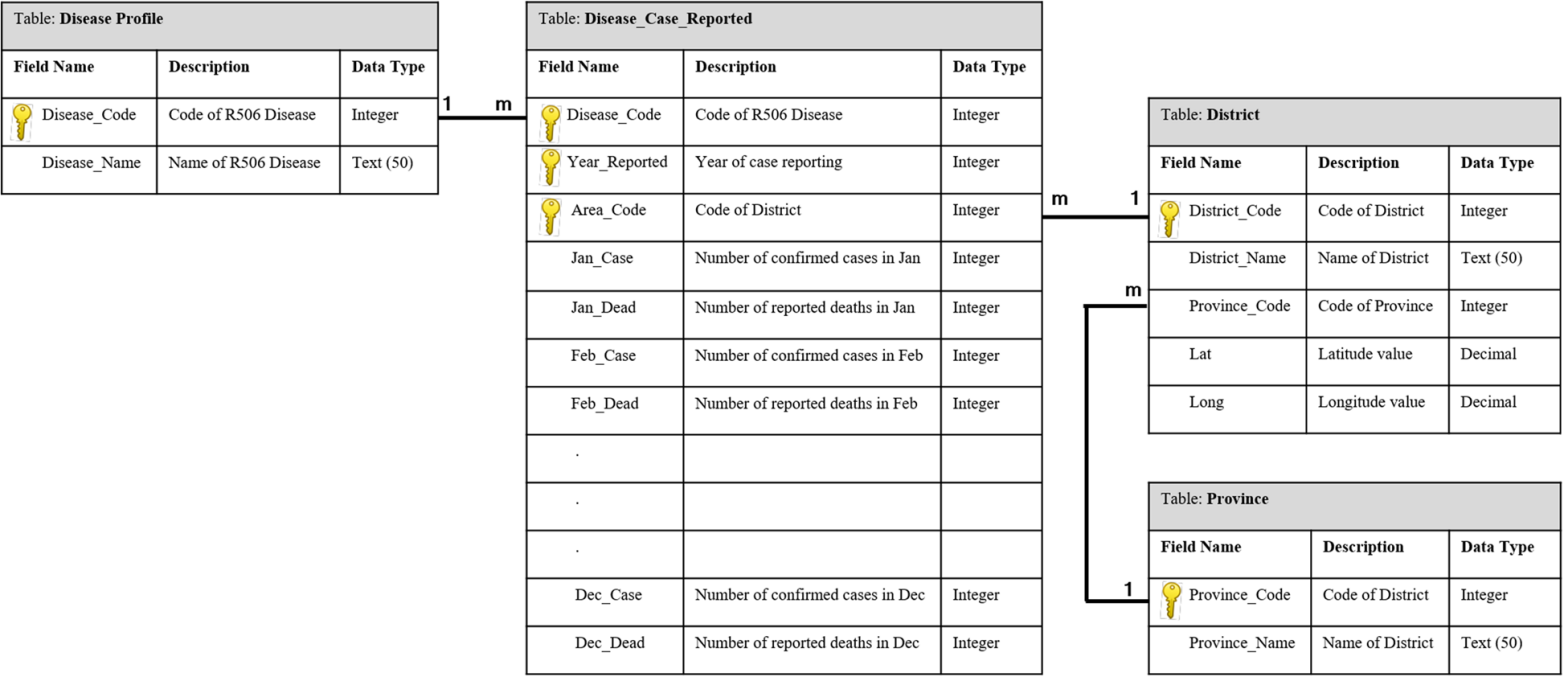
Main tables in the database used for storing R506 data

The user interface was designed based on the main user requirements obtained when applying the MADLC model (Fig. 1). The app conveyed information related to 14 diseases: dengue, malaria, influenza, food poisoning, rabies, cholera, leptospirosis, tuberculosis, chikungunya, diarrhea, hepatitis A, hepatitis B, scrub typhus, and typhoid. The R506 data from the national disease surveillance report published on the BoE website was downloaded every week, cleaned, and mapped to the appropriate fields before uploading to the database [22] (Figs. 2 and 3), which is hosted on a Firebase cloud computing platform. The app is capable of synchronizing the R506 data stored in the cloud database to the local SQLite database installed on every user’s smartphone.

**Fig. 4:**
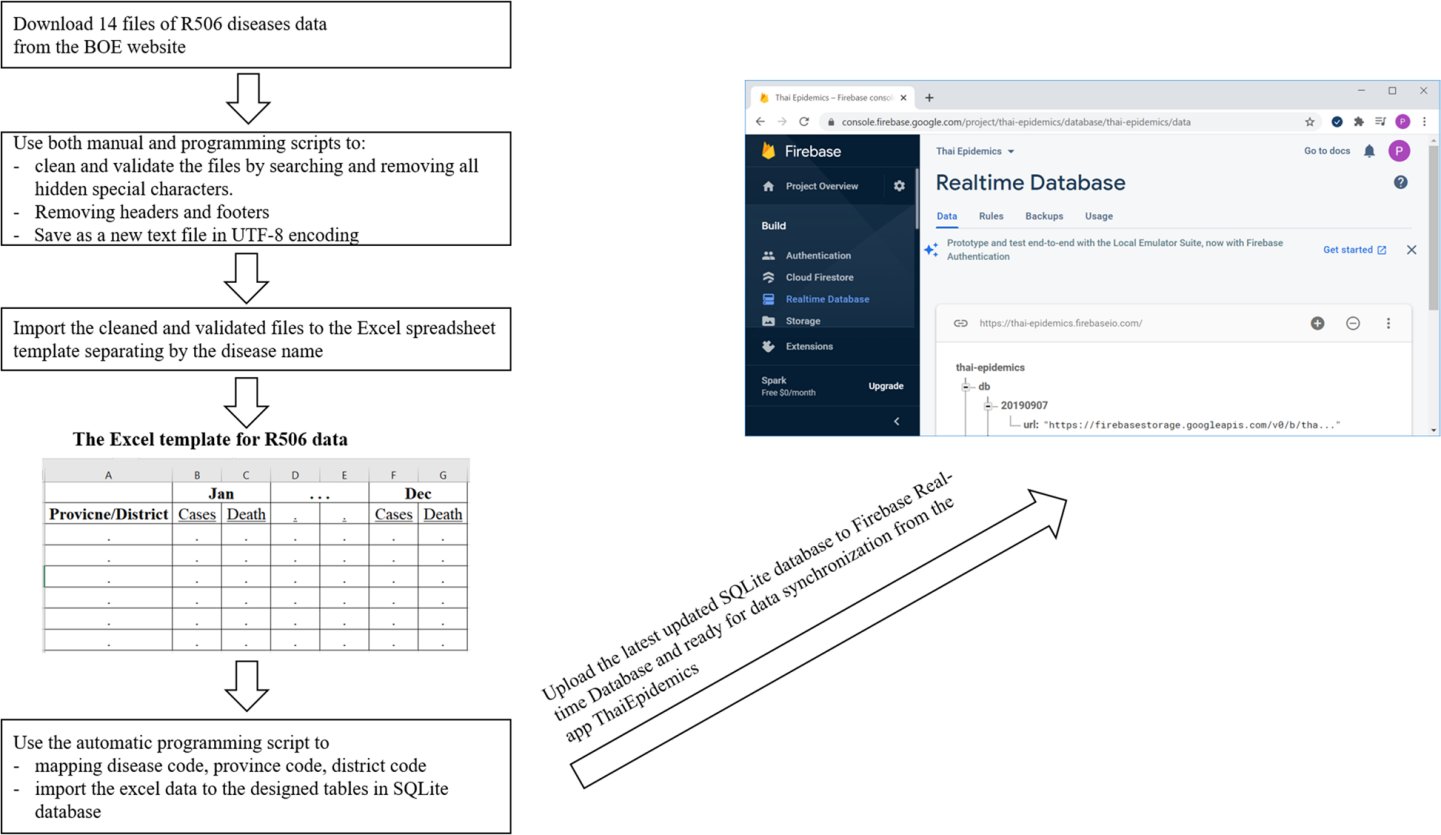
Steps of the downloaded R506 data preparation and transformation

The R506 reports published on the BoE website were not in a standard format for importing to the database. Thus, both automatic scripts and manual commands were created and used to manage the downloaded data files. The process of data transformation included preparing, cleaning, validating, and importing the validated, cleaned R506 data into the SQLite database before uploading to the Firebase real-time database hosted in the cloud for data synchronization to the mobile app (Fig. 4).

**Fig. 5:**
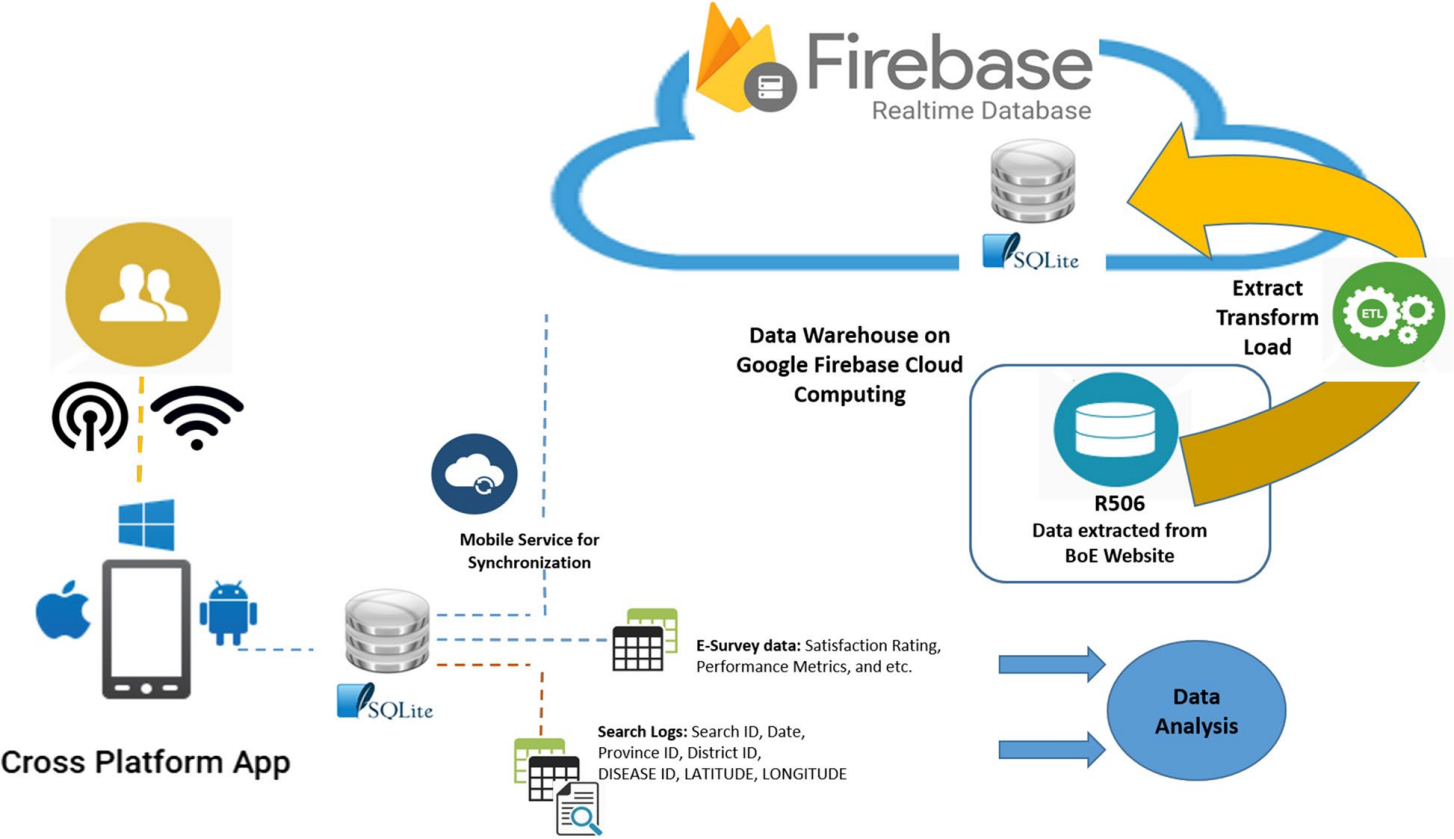
Conceptual diagram of the system and infrastructure

In addition, ThaiEpidemics stores the search logs recorded from user interactions and querying. First, the search logs were stored on the local mobile SQLite database before synchronizing to the Firebase database for further analysis. Similarly, the search log and feedback data collected from the e-survey were stored on the cloud database (Fig. 5).

### App displays and usage

**Fig. 6:**
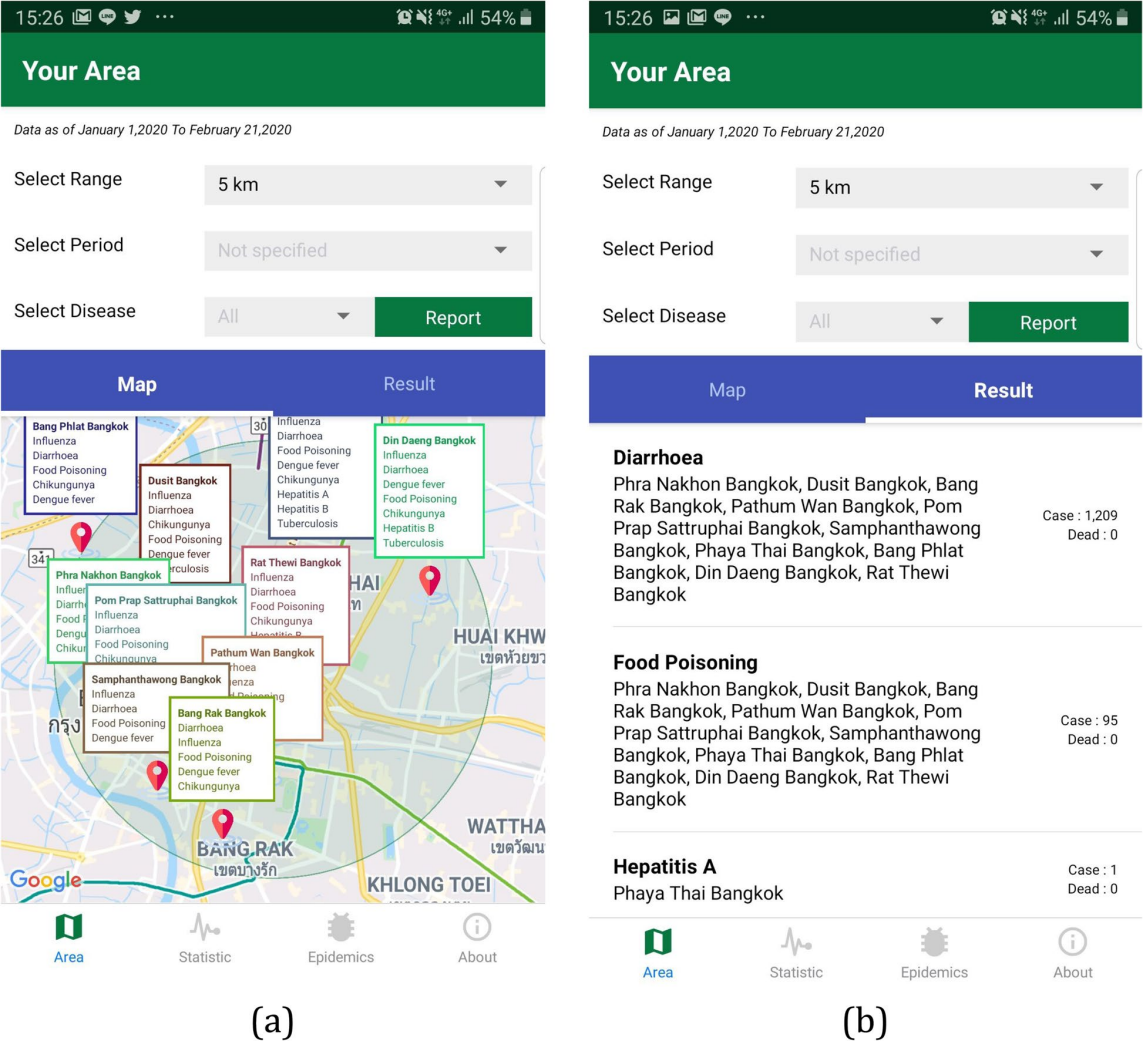
Screenshot of ThaiEpidemics: (**a**) disease prevalence and status around current location; (**b**) situation information categorized by disease name and district

**Fig. 7:**
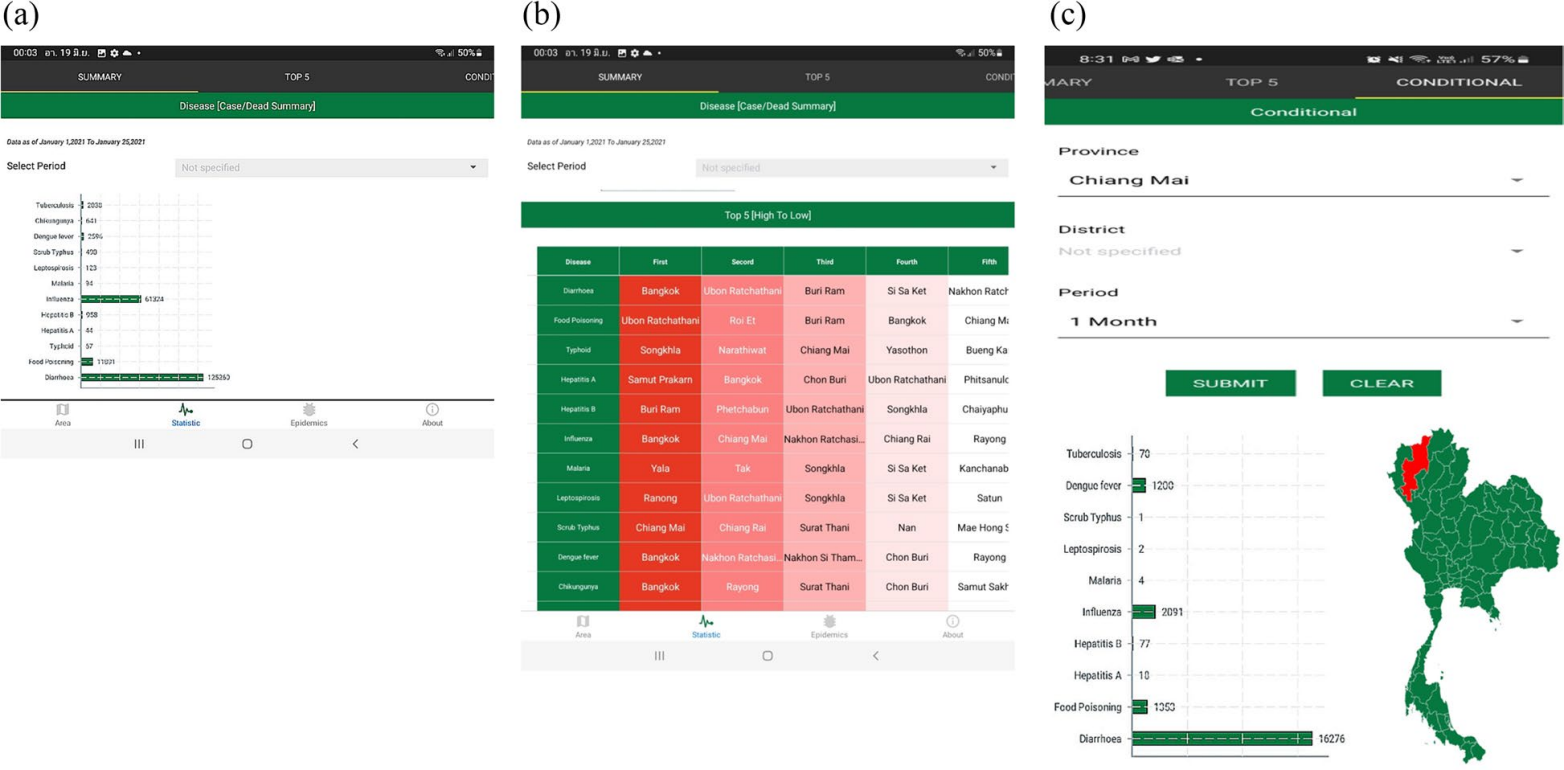
Screenshot of ThaiEpidemics at national-level visualization: (**a**) number of reported cases at the national level; (**b**) top five ranking of provinces with the number of cases of each disease; (**c**) specific queries for information about the disease prevalence and status in a particular area

ThaiEpidemics, was made available on both the Google Play and Apple App Stores. Participants used the app to obtain information about the disease prevalence and status in an area they planned to visit. There were three parameters: province, district, and period. The app searches for data in the database and then displays the information based on the entered parameters along with the location of the province on the map. The participants received the information and maps regarding disease prevalence and status s according to their queries (Figs. 6 and 7).

The parameters specified in the app were as follows: period or duration, distance or buffer radius from the current location, disease name, district, and province. Apart from searching for information about diseases around their current location, the participants could also search for information in areas they wanted to visit. When the participants checked the disease prevalence and status in an area, the app recorded the query in the database.

### App prototype testing

The app prototype was tested on travelers in tourist attractions in Bangkok and travelers seeking medical services at the Travel Clinic. We invited the participants to read a brochure about the mobile app, which contained the following information: objectives of the mobile app, main features and functions of the app, its benefits, screenshots of the app interfaces, platforms supported, and the app name for downloading from the Google Play or App Store. These stores are public, so anyone—not just individuals at the Travel Clinic—could search, download, and install the app on their smartphones. To ensure that only study participants could access the app, we included a password in the brochure for app installation. If someone were interested in the app and willing to participate in the installation and app usage, they could download the app and install it on their smartphone.

### App usability assessment

We evaluated user interaction employing a descriptive cross-sectional survey. We collected data from the participants from December 2019 to January 2020. We collected three types of data: user engagement (number of log-in times, how long travelers stayed in the app), search logs (disease name, province, district, duration), and answers to an e-survey. The search logs provided data about all the queries the participants entered on the app when searching for disease surveillance information for a particular area, date, or disease. The logs were stored in the local database before synchronization to the cloud database. The log contained the following data: date, province, district, disease, and location. We informed participants that the app would collect usage and search log details.

For the e-survey, an electronic questionnaire module was built into the app. Following the second log-in, the app prompted the travelers to complete the e-survey. The e-survey covered opinions and preferences regarding awareness of disease prevalence and status by specifying their level of agreement with prompted question using a five-point Likert scale. The questionnaire had two main parts: demographics and app satisfaction. The demographics section contained questions about the participants’ country of origin, gender, age, and education level. Items related to app satisfaction were derived with reference to a study of mobile health-care applications [23]. This part of the questionnaire was based on the use of the Mobile Application Rating Scale, which is a quality measurement tool for assessing the quality of health mobile apps [24].

### Statistical analysis

After the data collection was complete, descriptive statistics were utilized to analyze the characteristics of participants’ demographic data, including key variables of the three data sources. A chi-square test was used to examine the relationships among the following variables: improved awareness of disease prevalence and status, age-group, education level, and continent of origin. Binary logistic regression was also applied to determine whether the variables were significant predictors for improved awareness of disease prevalence and status after using the app.

## Results

### App utilization assessment

In all, 83 travelers downloaded and installed the app, and 52 of them responded to the e-survey. Table 1 presents the gender profile of the 52 respondents: 34 were male and 18 female. As indicated in the table, about 42% the respondents were under 30 years old and another 42% were between 31 and 50 years old. In terms of education, about 40% had attainment higher than a bachelor’s degree, while 35% had education lower than a bachelor’s degree. The respondents who competed the e-survey were those from different continents including approximately 44% Europeans, 29% Asians and 27% others.

**Table 1:** Characteristics of the 52 e-survey respondents

Characteristics	Frequencies *n* = 52)	Percentage
**Gender**		
Male	34	65.4%
Female	18	34.6%
**Age** (Years)		
< 30	22	42.3%
31 – 50	22	42.3%
> 51	8	15.4%
Min = 20 Max = 65 Mean = 35.96 SD = 11.71		
**Education Level**		
Lower than Bachelor	18	34.6%
Bachelor	13	25.0%
Higher than Bachelor	21	40.4%
**Continents**		
Asia	15	28.8%
Europe	23	44.2%
Others	14	26.9%

### Increasing disease infection awareness

Among the e-survey respondents, 92.3% believed that ThaiEpidemics had improved their awareness of the disease prevalence and status in Thailand and the areas of their itineraries, while 7.7% believed it did not. Table 2 presents the results of the chi-square test and binary logistic regression analysis. It was evident that gender, age-group, education level, and continent of origin were not significant predictors for increasing personal awareness of the disease infection by using the app.

**Table 2:** Results of the chi-square test and binary logistic regression analysis about whether the app could increase personal awareness of the disease prevalence and status

Characteristics	Total (*N* = 52)	Agreement if the app can leverage personal awareness of disease infection							
		Agree		Not Agree		Chi	95% CI for OR		
		N	Percentage	N	Percentage	*p*-value	OR	Lower	Upper
**Gender**	52	48	92.3%	4	7.7%	0.674			
Male *(ref)*	34	31	91.2%	3	8.8%		1		
Female	18	17	94.4%	1	5.6%		1.65	0.16	17.07
**Age**	52	48	92.3%	4	7.7%	0.356			
≤ 30 Yrs. *(ref)*	22	21	95.5%	1	4.5%		1		
31 – 50 Yrs.	22	19	86.4%	3	13.6%		0.30	0.03	3.15
≥ 50 Yrs.	8	8	100.0%	0	0.0%		–	–	–
**Education**	52	48	92.3%	4	7.7%	0.759			
Lower than Bachelor *(ref)*	18	16	88.9%	2	11.1%		1		
Bachelor	13	12	92.3%	1	7.7%		1.50	0.12	18.54
Higher than Bachelor	21	20	95.2%	1	4.8%		2.50	0.21	30.12
**Continent**	52	48	92.3%	4	7.7%	0.970			
Asia *(ref)*	15	14	93.3%	1	6.7%		1		
Europe	23	21	91.3%	2	8.7%		0.75	0.06	9.08
Others	14	13	92.9%	1	7.1%		0.93	0.05	16.42

### Search log analysis

The 83 participants logged in to the app 129 times. They submitted 281 queries (Table 3). Most participants (83.6%) did not specify a disease name when checking the situation around their current location: they wanted the app to show every disease reported within a specified radius. A few of them specified other diseases in their searches. As indicated in Table 4, there were 78 search logs related to future destinations.

**Table 3:** Checks for the disease prevalence and status from the participants’ current location

Name of Diseases searched by Travelers	Frequency (***n*** = 281)	Percentage %
ALL	235	83.6
Dengue Fever	12	4.3
Malaria	7	2.5
Influenza	6	2.1
Food Poisoning	5	1.8
Rabies	4	1.4
Other diseases	12	4.5

**Table 4:** Future destination searches

Destination searched by travelers	Frequency (***n*** = 78)	Percentage %
Bangkok	19	24.4
Chiang Mai	12	15.4
Phuket	8	10.3
Surat Thani	5	6.4
Krabi	4	5.1
Other provinces	30	38.8

### User engagement analysis

Table 5 shows the amount of time that participants spent using the app in a single session (app session length). In all, 31.8% used the app for up to 1 minute, 10.9% for up to 2 minutes, 11.6% for up to 3 minutes, 11.6% for up to 4 minutes, 10.9% for up to 5 minutes, and 23.3% used the app for over 5 minutes. The average (SD) time the participants spent using the app was 5.50 (10.6) minutes, while the median value was 3.0 minutes (minimum, 1 minute; maximum, 102 minutes). Table 6 shows the frequency and percentages for the number of times the participants used the app.

**Table 5:** App session length

How long the travelers used the app per visit login	Frequenc (***n*** = 129)	Percentage %
1 min	41	31.8
2 min	14	10.9
3 min	15	11.6
4 min	15	11.6
5 min	14	10.9
6 – 10 min	20	15.5
11 – 20 min	4	3.1
> = 21 min	6	4.7
Mean, 5.5 (SD, 10.6); Median, 3 (minimum, 1; maximum, 102)

**Table 6:** Number of times the participants used the app

Number of Login Times to the app	Number of Users (***n*** = 83)	Percentage %
1	60	72.3
2	12	14.5
> 3	11	13.2

## Discussion

A number of mobile apps have been developed using different tools to disseminate public health information to users [25–29]. As evident in the literature, disease is a risk that people identify as a key factor influencing their travel plans, as many individuals are concerned about getting sick while traveling abroad [30, 31]. We developed ThaiEpidemics for travelers to Thailand, with the aim to allow travelers to search for information and about the disease prevalence and status in the country. We intended the app to be useful for tourist to reduce their risk of disease exposure. ThaiEpidemics is similar to the CDC TravWell app, which is useful for international travel by providing generic information about global health safety information [25].

One study in Ontario found that situation awareness could be achieved from the data sources in disease surveillance reports [32]. In the present study, we developed ThaiEpidemics using national disease surveillance reports, which are updated weekly. Almost all our respondents who used the app to access health information believed that the app raised their awareness of diseases, regardless of the respondents’ education level, age, or continent of origin.

ThaiEpidemics uses two types of search logs. Logs concerning searches for tourist destinations, revealed that the top destinations: Bangkok, Chiang Mai, Phuket, Surat Thani, and Krabi, which is somewhat consistent with government reports [33, 34]. Search logs regarding disease information, revealed that most participants did not specify a disease. However, if the participants specified a type of disease, the logs clearly indicated that most requested information concerned dengue and malaria. This could reflect anxieties and vigilance regarding those illnesses. Findings are consistent with that of another study of travelers visiting Southeast Asia being at greater risk of dengue and malaria rather than chikungunya, Zika, and enteric fever [35]. It has been reported that 51% of travelers returning from Southeast Asia were sick with dengue [36], and that the number of returning travelers from that region had more dengue than malarial infections [37]. It has also been recorded that among Swedish travelers with dengue fever, 53% [38] and 49% [39] were infected in Thailand. One survey found that European travelers were concerned about the presence of mosquitoes or small insects in tourist accommodations owing to the fear of contracting diseases [40].

We identified one important issue regarding security concerns. Many individuals at the Travel Clinic were interested in the app and thought it would be helpful for their trips, however, only 83 wanted to install the app. While at the clinic, many travelers installed ThaiEpidemics, searched for information related to their itineraries, and then deleted the app. Smartphone users generally trust apps from well-known, official repositories, such as the Google Play and App Stores. A few studies reported that about 75% of smartphone users would download apps only from such repositories because doing so was secure [41, 42]. However, we found that though ThaiEpidemics was available on the Google Play and the App Stores, travelers still had security concerns about the app, and so they did not install it or removed it after immediate exploration. Their concerns were similar to those suggested in the literature about mobile security and privacy [43], collecting personal data, including GPS location [44], and consumption of resources [21]. One survey about smartphone users deciding whether or not to install an app reported that 60% decided not to do so when they discovered that the app required personal information, including the user’s GPS location; 43% deleted the app after downloading [45].

ThaiEpidemics has an important limitation: the timeliness of reporting. A major factor when providing public health information to travelers is the data source, which should be accurate and timely. Many mobile apps use data from social networks or search engines as a data source to generate reports in real time [25–27, 46]. Some apps employ the data reported by app users [47]. Such apps are based on official and unofficial data or both, leading to concerns about the accuracy and integrity of the data sources. ThaiEpidemics employs officially reported data. However, one drawback is that the data were not in real time (daily) as the BoE updated and published the R506 data on a weekly basis. We observed that most participants used the app just once while only a few logged in many times. It could be that the travelers deleted the app after the first or second log-in because they would be in Thailand only a few weeks and the data on the app were not updated frequently enough. It could also be that users decided to delete the app owing to higher data consumption, data security concerns, and GPS location identification.

### Study limitations & future directions

We developed ThaiEpidemics for foreign travelers in Thailand, and so the information related only to that country. The app covered only 14 diseases of concern to tourists, identified by travel medicine experts at the Travel Clinic. With the app’s table and database design, disease data other than R506 could be incorporated. Thus, ThaiEpidemics could be expanded to include other disease information and for other locations. ThaiEpidemics was developed as an open-source software; it discloses and shares the source code, allowing developers to learn, modify, or use it under the terms of open-source licenses, allowing it to be adapted to suit other conditions [48]. ThaiEpidemics was created using React Native as a developing tool, which is a popular open-source format in GitHub [49]. All developers are able to update or extend their existing source code to enhance and expand the use of open-source software at no cost.

Another study limitation was that the assessment of the app’s usability was based on a small sample of 83 participants. The outbreak of Covid-19 during data collection posed a major problem to this study, including the Thai government issuing restrictions on foreign travelers entering the country. Consequently, the number of tourists to Thailand has been decreasing, and only a few visitors went to the travel clinic.

From a development aspect, ThaiEpidemics used data from the BoE website as its data source, but the data report was in Rich Text Format. Accordingly, there was a tendency for data error or corruption when transforming to a standard database. Therefore, in the data preparation process, we manually downloaded the report file, validated and converted it to a text file, and finally used a programming script to import the cleaned text file data into the SQLite database before uploading to the Firebase database. This platform offers a real-time database, which allows mobile apps to store data. ThaiEpidemics synchronized, read, processed, and displayed disease information and situation for mobile devices.

To achieve long-term sustainability, mobile app developers need to be mindful of the system development life cycle. After deployment, an app must be maintained regularly. Making a mobile app more sustainable is very challenging due to ongoing updates of the mobile OS and changes in user requirements. To grasp the requirements for the future direction of an app, the developer has to collect user feedback and analyze usage. To make ThaiEpidemics sustainable, the cooperation of the BoE in preparing the R506 dataset in a form that can be easily migrated is necessary. ThaiEpidemics could be more successful with the collaboration of other government agencies and authorities to help promote it and build trust among users regarding data security and confidentiality. Promoting ThaiEpidemics could be undertaken through the websites or social media of travel agencies and medical providers, such as the Tourism Authority of Thailand and several travel clinics.

## Conclusions

Using cross-platform technology, ThaiEpidemics was developed for international travelers to Thailand. The app provides users with functions and features to access information about the disease prevalence and status in the country. Our participants could obtain information around their current location and other locations in Thailand. We tested the prototype of our app among visitors to the Travel Clinic at the Hospital for Tropical Diseases. Most of the travelers we approached were interested in the app, which allowed them to receive useful information about the disease situation in places they intended to visit. 83 people installed the app, and among those who did not, major concerns were related to privacy and security issues. It was found that most participants believed that receiving disease information about their current and future destinations was very useful, and that the app raised their awareness about health issues. Most users conducted searches for the 14 major travel diseases installed in the app that are of concern to travelers, while some searched specifically for malaria and dengue. ThaiEpidemics was developed using open-source software. General developers and others can use the files for further analysis and to build reports and dashboards to meet their own requirements. As the app showed its potential usefulness for the tourists, we hope to deploy the app again after the country relaxes the current restrictive tourism policies, in 2022. Expanded from the basic 14 diseases of concern to tourists coming to Southeast Asia, the app would include additional information on the COVID-19 disease situation.

## Data Availability

The data collected during the current study are available from the corresponding author on reasonable request.
